# Characterization of the first complete chloroplast genome sequence of *Celtis sinensis* (Cannabaceae) and its phylogenetic implications

**DOI:** 10.1080/23802359.2019.1692705

**Published:** 2019-11-18

**Authors:** Guozheng Wang, Yunyan Zhang, Jiehan Zhou, Mengyuan Zhang, Yimin Hu, Yongjing Tian, Jingbo Zhou, Zhongsheng Wang

**Affiliations:** a College of Life Sciences, Nanjing University, Nanjing, China;; b Department of Forest Resources, Anhui Academy of Forestry, Hefei, China

**Keywords:** *Celtis sinensis*, resistant plant, plastid genome, phylogenomics

## Abstract

*Celtis sinensis* Pers. is a popular garden landscape tree in riparian areas and a valuable resistant tree in many extreme environments. Here, we determined the first complete chloroplast genome of *C. sinensis* using high-throughput sequencing technology. Our results showed the chloroplast genome of *C.sinensis* was 159,092 bp long and displayed a typical quadripartite structure consisting of a pair of inverted repeats with a length of 26,895 bp and separating by two single-copy regions (LSC, 86,085 bp and SSC, 19,217 bp). Besides, the chloroplast genome of *C. sinensis* totally contained 131 genes, including 87 protein coding genes, 36 transfer RNAs and eight ribosomal RNAs. Additionally, a maximum likelihood phylogenetic analysis based on the 19 chloroplast genomes demonstrated the monophyly of Cannabaceae and *C. sinensis* formed a sister clade to *Celtis biondii*.

*Celtis sinensis* Pers., a deciduous tree native to Japan, Korea and China (Panetta 2001), belongs to the genus *Celtis* within the eudicot family Cannabaceae *sensu* APG IV (The Angiosperm Phylogeny Group 2016). It is widely cultivated as a popular garden landscape tree in riparian areas because of its characteristics of elegant dense shade, hard trunk and fast growth speed (Siebert et al. 2018). Besides, it performs strong resistance to various adverse conditions, such as saline-alkali soil, industrial pollution areas and coastal inland transition zones (Jim and Liu 2001; Bhuju and Ohsawa 1999). Previous studies about *C. sinensis* mainly focused on its community structure, reproductive regeneration mechanism and ecological adaptability (Lee et al. 2004; Zhang et al. 2016; Siebert et al. 2018). However, researches regarding its genetic backgrounds are extremely scarce. Fortunately, with the advent of next-generation sequencing technologies, abundant genomic resources of our interested species have been produced in a rapid and cost-effective way in recent years (Hao et al. 2011; Malé et al. 2014). To comprehensively understand the genetic background of *C. sinensis* and facilitate its utilization, in this study, we characterized the first complete chloroplast genome sequence of *C. sinensis* based on the Illumina pair-end sequencing data and registered it into the GenBank with the accession number MN481989.

The silica-gel dried leaves of *C. sinensis* were sampled from Mo Mountain (China; 34°30.8589′N, 117°18.2520′E) and the voucher specimen (accession number: ZYY190729) was deposited at the Herbarium of Zhejiang University (HZU). Total genomic DNA was isolated with a modified CTAB method (Doyle and Doyle 1987). Subsequently, paired-end sequencing library was constructed and sequenced on the Illumina HiSeq X-Ten platform at Beijing Genomics Institute (Shenzhen, China). In results, approximately 3.5 Gb of raw data were generated and we de novo assembled the complete chloroplast genome using GetOrganelle pipeline (https://github.com/Kinggerm/GetOrganelle). The genome was then automatically annotated using CpGAVAS (Liu et al. 2012), with manual adjusting and confirming the annotated genes in Geneious v11.0.4 (http://www.geneious.com/; Kearse et al. 2012).

The complete chloroplast genome of *C. sinensis* was 159,092 bp in length, displaying a typical circular quadripartite structure with a pair of inverted repeat regions (IR, 26,895 bp) separated by a large single-copy region (LSC, 86,085 bp) and a small single-copy region (SSC, 19,217 bp). It encoded 112 unique genes, including 78 protein-coding genes, 30 tRNA genes and four rRNA genes. Nine protein-coding genes (*rps*16, *atp*F, *roc*1, *pet*B, *pet*D, *rpl*16, *rpl*2, *ndh*B, *ndh*A) and six tRNA gene (*trn*K-UUU, *trn*G-UCC, *trn*L-UAA, *trn*V-UAC, *trn*L-GAU, *trn*A-UGC) contained a single intron, while three protein-coding genes (*ycf*3, *clp*P, *rps*12) possessed two introns. The gene *rps*12 was trans-spliced; with the 5′-end exon located in the LSC region and two copies of 3′-end exon and intron in the IR regions. The GC content of the total chloroplast genome was 36.3%, with the corresponding values of 34.1, 29.8 and 42.3% for the LSC, SSC and IR regions, respectively.

Furthermore, to ascertain phylogenetic position of *C. sinensis* in the family Cannabaceae, we selected 16 published plastomes of Cannabaceae and the three taxa of Ulmaceae as outgroups, employing the GTR + R + I model and 1000 bootstrap replicates under the maximum-likelihood (ML) inference in RAxML-HPC v.8.2.10 on the CIPRES cluster (Miller et al. 2010). Our reconstructed phylogenetic tree strongly supported the monophyly of Cannabaceae and *C. sinensis* was sister to *Celtis biondii* with full bootstrap support (Figure 1). These novel genomic resources and plastid phylogenomics here will largely enrich the genetic resources and enhance the molecular breeding of *C. sinensis*.

**Figure 1.**
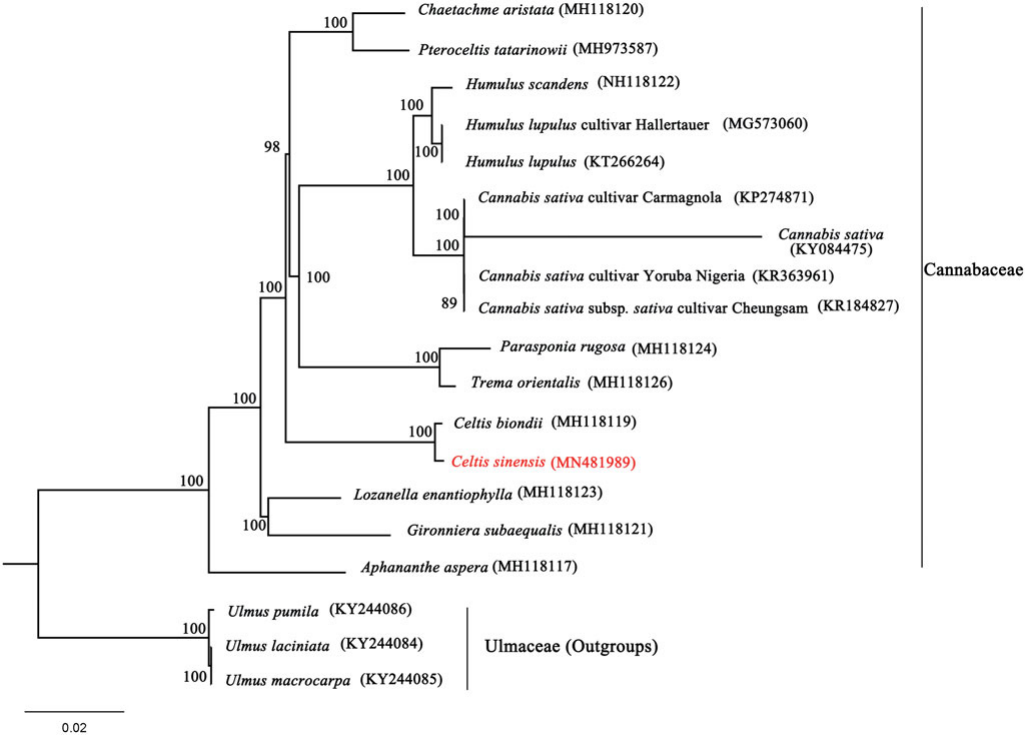
The phylogenetic tree based on 19 complete chloroplast genome sequences. Relative branch lengths are indicated. Numbers near the nodes represent ML bootstrap values.
